# Chagas disease: a tropical infection of interest to the radiologist

**DOI:** 10.1590/0100-3984.2016.49.6e1

**Published:** 2016

**Authors:** Edson Marchiori

**Affiliations:** 1 Full Professor of Radiology at the Universidade Federal do Rio de Janeiro (UFRJ), Rio de Janeiro, RJ, Brazil. E-mail: edmarchiori@gmail.com.

This issue of **Radiologia Brasileira** features an interesting study on the radiological findings observed in patients with megaesophagus secondary to Chagas disease (CD), as identified on chest X-rays and esophagograms^(1)^. The first point to be emphasized about that study is that, although it was conducted recently, it employed conventional radiology. Although many think otherwise, conventional radiographs continue to play a critical role in the investigation of thoracic diseases. When performed and interpreted appropriately, conventional radiology provides useful information for the characterization of lesions, in some cases being the only complementary imaging modality required. However, conventional radiographs have certain limitations, which often makes it necessary to use other imaging methods, computed tomography in particular^(2)^. Another relevant aspect of the study in question is that it dealt with a disease that has a high incidence and prevalence in Latin America, especially in Brazil. Although other tropical infections, such as paracoccidioidomycosis, have been the subject of various recent publications in the radiology literature of Brazil^(3-7)^, the radiological evaluation of CD has been neglected by researchers in the country. Even for paracoccidioidomycosis, the volume of radiological publications has been considered insufficient, given the importance of the disease. In a recent editorial, Rodrigues**(**^8)^, referring to the lack of studies on the imaging aspects of paracoccidioidomycosis, pointed out that this "void" in the literature is largely our fault (i.e., that of researchers working in Brazil), because Brazil is the country where the disease is most common. The author also underscored the importance of studies on these types of diseases, which are typical and endemic in our country, for improving the understanding of their characteristics, thus increasing the accuracy of their diagnosis, as well as the appropriateness of the treatments instituted.

Also known as American trypanosomiasis, CD is caused by the protozoan *Trypanosoma cruzi*, which has a high prevalence and causes significant morbidity in Latin America. Although the incidence of CD has been declining in the last decades, an extremely high number of patients still suffer from this disease. The majority of patients with chronic CD are elderly. That is because most CD patients come from areas in which the vector was eradicated three or four decades ago. Approximately 30% of individuals chronically infected with *T. cruzi* develop cardiac abnormalities, whereas approximately 10% develop digestive, neurological, or mixed disorders, all of which require specific treatment. Among the aspects of CD that can be assessed by imaging methods, the main cardiac alteration is chronic heart disease, whereas the main alterations in the digestive tract are dilatations, such as megaesophagus and megacolon^(9)^.

Cardiac involvement is the main determinant of the prognosis of CD. Cardiac magnetic resonance imaging (MRI) has been the examination of choice for evaluating the heart in CD patients. Cardiac MRI is noninvasive, does not use ionizing radiation, and provides high resolution images, allowing anatomy and function to be assessed, as well as allowing tissues to be characterized. New techniques have come to be routinely used for detailed assessment of cardiac function in CD, such techniques including myocardial tagging; high-resolution cine-MRI; late myocardial enhancement (for detection of myocardial fibrosis); myocardial perfusion; techniques for the detection of inflammation and edema; and techniques that facilitate the monitoring of stem cell injections for the treatment of CD-related cardiomyopathy^(9)^.

In relation to digestive tract impairment, idiopathic achalasia and CD-related achalasia are similar, in terms of their clinical and radiological manifestations, and require the same treatment. The term achalasia refers to the motor disease of the esophagus, and megaesophagus refers to its consequence. Megaesophagus is one of the clinical forms of CD that, albeit benign, is chronic and progressive, thus having relevant clinical repercussions. The most common symptom is dysphagia, followed by regurgitation, heartburn, and chest pain. The dysphagia is progressive, evolving over the course of several years. Patients with megacolon secondary to CD present severe, prolonged constipation for years or decades. Such patients often take mineral oil for the treatment of chronic constipation. Therefore, lipoid pneumonia is a potential complication of CD. In addition, achalasia is a risk factor for aspiration, also promoting the development of lipoid pneumonia^(10-12)^.

In conclusion, CD should be included in the differential diagnosis of patients presenting with altered digestive tract motility, severe dysphagia, or constipation, with or without cardiac involvement.
